# Rapamycin prevents endothelial cell migration by inhibiting the endothelial-to-mesenchymal transition and matrix metalloproteinase-2 and -9: An in vitro study

**Published:** 2011-12-24

**Authors:** Hua Gao, Jingjing Zhang, Ting Liu, Weiyun Shi

**Affiliations:** 1 State Key Lab Cultivation Base, Shandong Provincial Key Lab of Ophthalmology, Shandong Eye Institute, Qingdao, China; 2 Qingdao University, Qingdao, China

## Abstract

**Purpose:**

To evaluate the influence of rapamycin on endothelial-mesenchymal transition and matrix metalloproteinase (MMP) secretion by human umbilical vein endothelial cell line EA.hy926 and explore rapamycin’s angiogenesis inhibition mechanism.

**Methods:**

EA.hy926 cells were cultivated in vitro. After the cells attained complete confluency, an artificial scratch was made through the monolayer with a sterile plastic 100 μl micropipette tip. Cell morphology changes were observed. The expression of vascular endothelial (VE)-cadherin, vimentin, and Twist protein were examined by immunofluorescence. After scratching, the cells were treated with 10, 100, and 1,000 ng/ml rapamycin for durations of 24, 48, and 72 h. Cell proliferation was then assessed using methyl thiazolyl tetrazolium assay. Cell migration ability was examined, and the expression of VE-cadherin, vimentin, and the Twist transcription factor in mRNA levels was evaluated with reverse transcriptase PCR. The expression of gelatinases (MMP-2 and MMP-9) was examined using gelatin zymography.

**Results:**

After scratching, the endothelial cells were able to migrate via an endothelial-to-mesenchymal transition, which was related to Twist expression. Finally, mesenchymal cells transitioned into endothelial cells and reached cell confluency again. The growth of EA.hy926 cells was not affected by rapamycin concentrations of 10 ng/ml or 100 ng/ml during treatment periods of 1, 2, and 3 days; however, cell growth was inhibited by 1,000 ng/ml rapamycin with a three-day treatment period. Rapamycin successfully inhibited cell migration at concentrations of 10 ng/ml, 100 ng/ml, and 1,000 ng/ml for a treatment period of up to 8 h. Different concentrations of rapamycin induced the expression of VE-cadherin, inhibited vimentin and Twist expression in the endothelial cells, and inhibited endothelial cell secretion of MMP-2 and MMP-9.

**Conclusions:**

Rapamycin inhibited cell migration and extracellular matrix degradation by inhibiting endothelial-to-mesenchymal transition and the endothelial cell secretion of MMP-2 and MMP-9; these may be possible mechanisms for the inhibition of angiogenesis by rapamycin.

## Introduction

Neovascularization is a complex process and is tightly regulated by many positive and negative factors [1-5]. Endothelial cell migration plays an important role in angiogenesis [6]. Rapamycin is an immunosuppressive macrolide. Its strong immune inhibition effects, as well as its ability to effectively inhibit corneal neovascularization and tumor angiogenesis [4,7], have gained much attention. Rapamycin has effectively inhibited angiogenesis by inhibiting endothelial cell migration and proliferation [4]. However, very little is known about the mechanism by which rapamycin inhibits endothelial cell migration. This might occur by the direct suppression of mammalian target of rapamycin (mTOR) expression [4] and reduction of vascular endothelial growth factor (VEGF) expression [8] or through some other mechanism. The endothelial-to-mesenchymal transition (EndoMT), whereby endothelial cells can transdifferentiate into mesenchymal cells accompanied by decreased endothelial markers (vascular endothelial [VE]-cadherin) and increased mesenchymal markers (vimentin), is an important step of angiogenesis during embryo development [9,10], as well as in kidney fibrosis and other fibrotic diseases [11,12]. It is still not clear whether rapamycin inhibits endothelial cell migration by inhibiting EndoMT. Kwon found that rapamycin had not affected the expression of MMP-9 mRNA (mRNA) in the alkaline-burned cornea [4], but many reports have also found that rapamycin may suppress the expression of MMPs [13-15]. Therefore, this study focused on whether EndoMT occurred during the cell migration process, and whether rapamycin inhibited endothelial cell migration by inhibiting EndoMT and blocking the production of MMP-2 and MMP-9.

## Methods

### Materials

An EA.hy926 cell line was generously provided by Cora-Jean S. Edgell from the University of North Carolina at Chapel Hill and the Tissue Culture Facility in the United States. Additional materials included fetal bovine serum (FBS; Gibco Company); goat polyclonal antibodies specific for human Twist (Santa Cruz Company, Santa Cruz, CA); and mouse monoclonal antibodies (Mabs) binding to the human cell type–specific protein VE-cadherin (Santa Cruz Company). Rabbit polyclonal antibodies specific for human vimentin, fluorescein isothiocyanate (FITC)-labeled goat antirabbit IgG antibody, FITC-labeled rabbit antigoat IgG antibody and tetramethyl rhodamine isothiocyanate (TRITC)-labeled goat antimouse IgG antibody were from Zhongshan Golden Bridge Biotechnology Co. Ltd. (Beijing, China). Primers were purchased from the Invitrogen Corporation (China). Rapamycin was provided by the North China Pharmaceutical Group New Drug Research and Development Center. All chemical reagents were of analytical grade and were purchased from Sigma.

### Cell cultures

The EA.hy926 cell line, a permanent endothelial cell line derived from human umbilical vein endothelial cells (HUVECs) by fusion with the lung carcinoma cell line A549, were maintained in Dulbecco’s Modified Eagle’s Medium (DMEM)-high glucose with 4500 mg/l glucose (Gibco, Carlsbad, CA), supplemented with 10% FBS at 37 °C in a 5% CO_2_ incubator. When the experiment was conducted, EA.hy926 cells were seeded in tissue culture plates and maintained in DMEM-high glucose with 4500 mg/l glucose, supplemented with 2% FBS. A 5 mg/ml rapamycin stock solution was prepared in dimethyl sulfoxide (DMSO; Xinxing Chemicals Company, Panjin, China) and stored at −20 °C. The culture medium was replaced with rapamycin-containing medium (10, 100, and 1,000 ng/ml rapamycin) or vehicle control medium (0.1% DMSO in fresh medium).

### Endothelial cell morphology and immunofluorescence staining

EA.hy926 cells were seeded in 96-well tissue culture plates in amounts of 5×10^3^ cells/well. After complete confluency, an artificial scratch approximately 300 μm wide was made through the monolayer with a sterile plastic 100 μl micropipette tip. After 24 h, 48 h, and 72 h of incubation, cell morphology changes were observed under inverted microscope. The expression of VE-cadherin, vimentin, and Twist protein was examined using immunofluorescence staining. Primary antibodies were VE-cadherin mouse antihuman, vimentin rabbit antihuman, and Twist goat antihuman; secondary antibodies were FITC-labeled goat antirabbit IgG, TRITC-labeled goat antimouse IgG antibody, FITC-labeled rabbit antigoat IgG, and nucleus stained with Hoechst (Beyotime Institute of Biotechnology, Shanghai, China). Finally, the cells were visualized and photographed with an Olympus fluorescence microscope (Olympus BX60, Tokyo, Japan).

### Cell proliferation analysis

The cells (5×10^3^) were inoculated in 96-well plates (150 µl/well), incubated for 12 h, and serum-starved overnight. Cell proliferation was measured using 3-(4, 5)-dimethylthiahiazo (-z-y1)-3, 5-di- phenytetrazoliumromide (MTT) [16].The cells were separately treated with 10, 100, and 1000 ng/ml rapamycin for durations of 24, 48, and 72 h, with six wells for each group. The cells of the control group were left untreated. 3-(4,5)-dimethylthiahiazo (-z-y1)-3, 5-di- phenytetrazoliumromide (MTT, Sigma; 5 g/l) was used as an incubation solution, and the wells were incubated for 4 h. DMSO (150 µl) was added to the culture medium with low-speed oscillation for 10 min. The optical density (*A* value) was measured at 492 nm with a continuous spectrum densitometer (Spectra Max M2; Molecular Devices, Sunnyvale, CA).

### Cell migration ability examination

The cells (5×10^3^) were inoculated in 96-well plates (150 µl/well). An artificial scratch was performed after complete confluency and separately treated with 10, 100, or 1000 ng/ml rapamycin for a duration of 8 h. The cells of the control group were left untreated. Photographs of treated cells within the scratch were taken with a phase-contrast microscope (magnification of 100×; Nikon Diaphot 300; Nikon, Tokyo, Japan) connected to a digital camera. We randomly selected six photographs and counted the cells that had moved into the wounded area in the selected photographs.

### Evaluation of the gene expression of vascular endothelial-cadherin, vimentin, and Twist

The cells (500×10^3^) were inoculated in 6-well plates (2 ml/well). After reaching confluency, the cells were randomly divided into three groups: (1) the no-scratch group, (2) the scratch group, and (3) the rapamycin group was further divided into three groups: after being artificially scratched, the cells were separately treated with 10, 100, and 1,000 ng/ml of rapamycin for an incubation of 24 h. The cells were collected by centrifugation and the total RNA was extracted with the NucleoSpin RNA II System (Company Macherey-Nagel, Düren, Germany) according to the manufacturer’s protocol. PCR primers were designed on the basis of the published human gene sequences (Table 1). Reverse transcriptase–PCR was performed to evaluate the expression of these genes in EA.hy926 cells using the housekeeping gene glyceraldehyde-3-phosphate dehydrogenase (*GAPDH*) as an internal control [17].

**Table 1:** PCR primers of human gene sequences

**Gene name**	**Primer sequence (5′-3′)**	**Gene size**
VE-cadherin [40]	F: AACTTCCCCTTCTTCACCC	387 bp
	R: AAAGGCTGCTGGAAAATGA	
Vimentin [41]	F: GGCTCAGATTCAGGAACAGC	327 bp
	R: GCTTCAACGGCAAAGTTCTC	
Twist [42]	F: GGAGTCCGCAGTCTTACGAG	201 bp
	R: TCTGGAGGACCTGGTAGAGG	
*GAPDH* [17]	F: ACCACAGTCCATGCCATCAC	439 bp
	R: TCCACCACCCTGTGGCTGTA	

Abbreviations: *GAPDH* represents glyceraldehyde 3-phosphate dehydrogenase, VE represents vascular endothelial.

### Gelatin zymography

The endothelial cells of each group were incubated according to the protocol used for reverse transcriptase–PCR. The culture supernatants were harvested at 24 h and mixed with a gel sample buffer (0.5M Tris-HCl, glycerol, 10% sodium dodecyl sulfate [SDS], β-mercaptoethanol, and 0.5% bromophenol blue). Ten micrograms of protein were taken by SDS PAGE (PAGE) separation; the SDS–PAGE gels contained 0.1% gelatin (Sigma) [18]. After electrophoresis, the gels were washed in 50 mM Tris buffer containing 2.5% Triton X-100. The gels were incubated for an additional 18 h in incubation fluid (50 mM Tris buffer [pH 7.6], 10 mM CaCl_2_, and 200 mmol/l NaCl). The gels were stained with 0.5% Coomassie blue that contained 30% methanol and 10% glacial acetic acid and subsequently destained in 45% methanol/10% acetic acid/H_2_O. White bands on a blue background indicated zones of digestion corresponding to the presence of different MMPs. Gels were scanned, and a density analysis of the bands was performed using ImageJ software.

### Statistical analysis

All values in the figures and text were expressed as mean±standard deviation (SD) of three observations. All data were analyzed with the SPSS 11.5 statistical package. A one-way analysis of independent samples *t*-test for two-sample comparisons was used to compare the means of two groups. A p value less than 0.05 was considered statistically significant. The reported results were representative of three independent experiments.

## Results

### Changes in endothelial cell morphology during migration and expression of vascular endothelial cadherin, vimentin, and Twist

**Figure 1:**
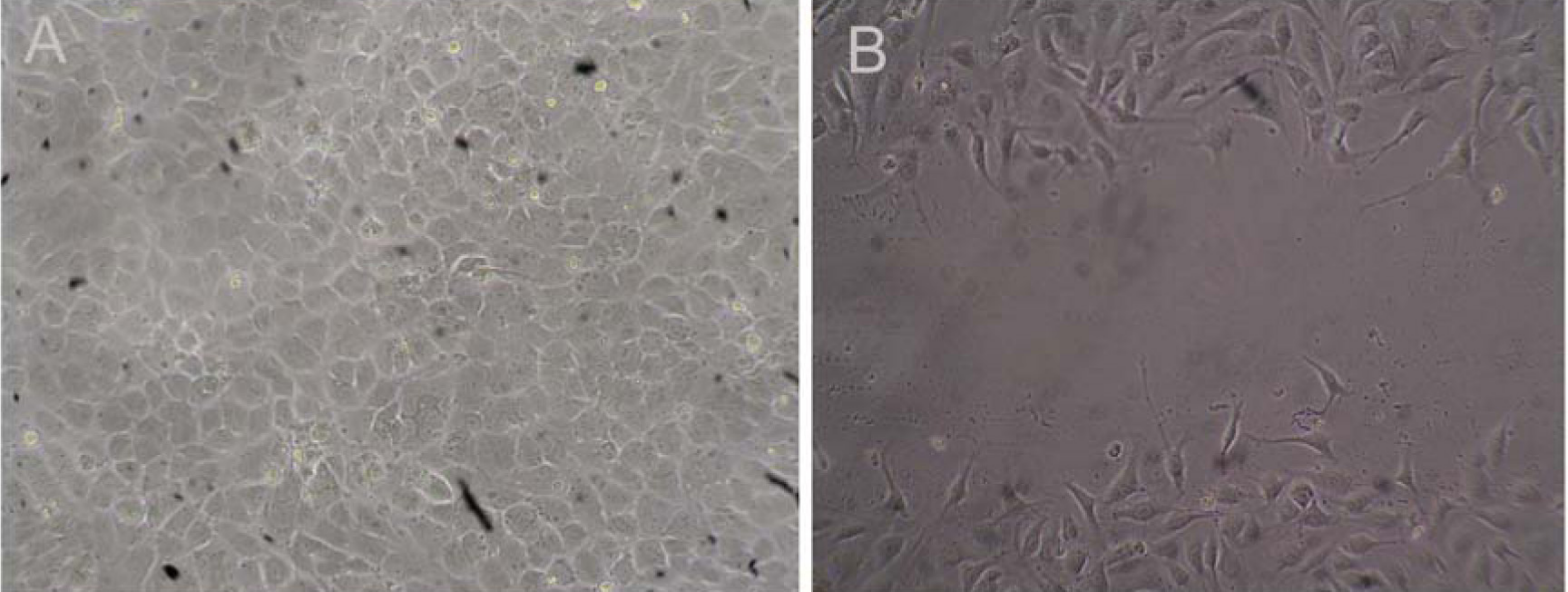
Cell morphology. **A**: In the no-scratch group, after cell confluency, EA.hy926 cells formed a cobblestone-like monolayer. **B**: In the scratch group, the endothelial cells that migrated into the scratch wound showed a fibroblast-like morphology with visible pseudopods (contrast phase microscope 100×).

**Figure 2:**
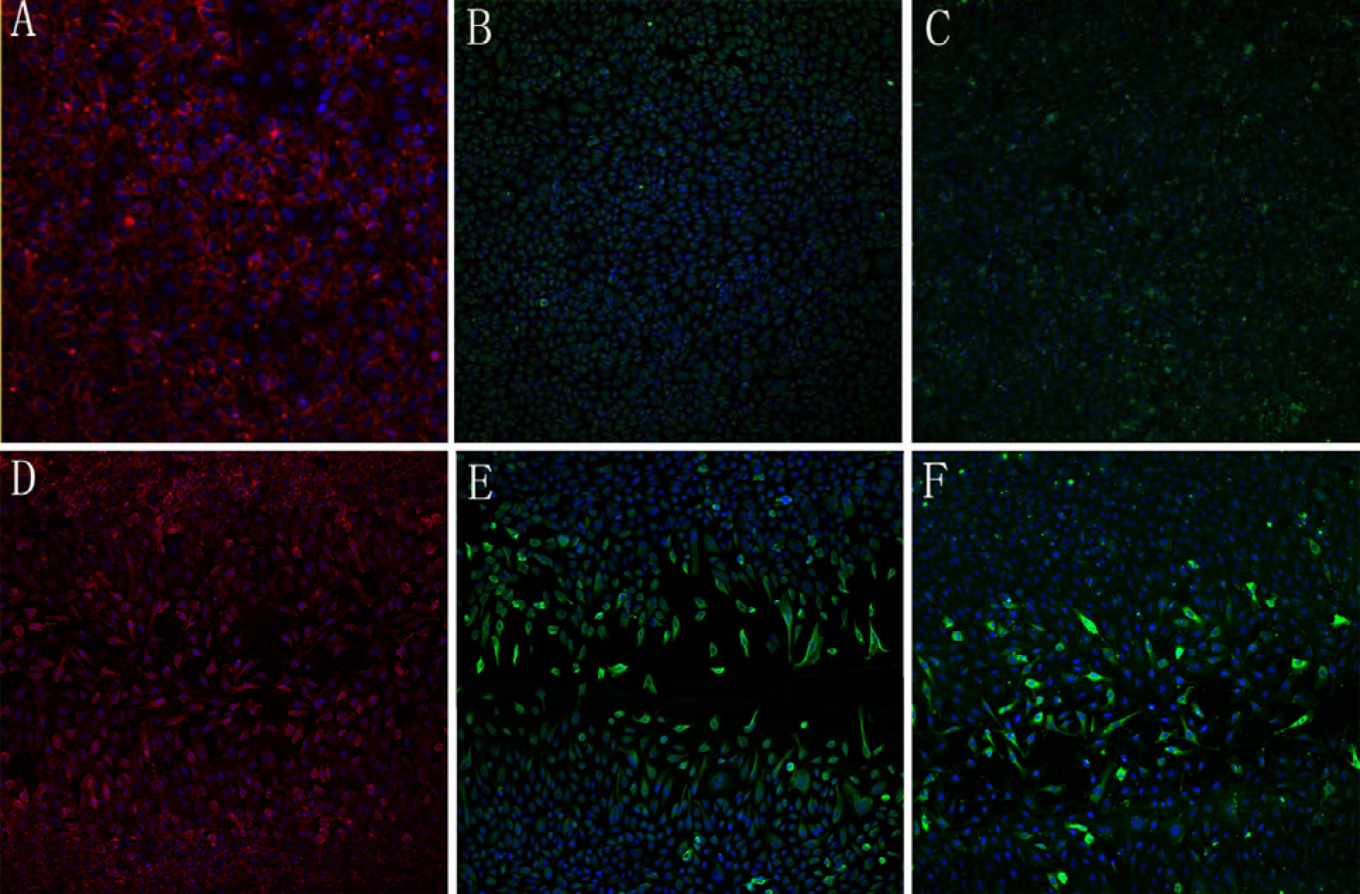
Immunofluorescence images. **A**: In the no-scratch group, after cell confluency, vascular endothelial (VE)-cadherin yielded continuous, linear staining around the periphery of EA.hy926 cells (confocal microscopy 200×). **B**: In the no-scratch group, after cell confluency, there was no expression of vimentin protein (confocal microscopy 100×). **C**: In the no-scratch group, after cell confluency, there was no expression of the Twist protein (confocal microscopy 100×). **D**: In the scratch group, the VE-cadherin expression of the endothelial cells that migrated into the scratch wound was decreased at endothelial cell-cell junctions and was mainly localized to the cytoplasm; VE-cadherin yielded continuous, linear staining around the periphery of EA.hy926 cells on the outside of the scratch wound (confocal microscopy 100×). **E**: In the scratch group, the vimentin expression of endothelial cells that migrated into the scratch wound (confocal microscopy 100×). **F**: In the scratch group, the Twist expression of endothelial cells that migrated into the scratch wound (confocal microscopy 100×).

After cell confluency, EA.hy926 cells ultimately formed a cobblestone-like monolayer (Figure 1A). Antibodies that recognized VE-cadherin yielded continuous, linear staining around the periphery of EA.hy926 cells (Figure 2A), but there was no expression of Twist protein (Figure 2B) or vimentin protein (Figure 2C).There was a striking change in the morphology of the endothelial cells that migrated into the scratch wound, which developed a fibroblast-like morphology with visible pseudopods (Figure 1B); VE-cadherin expression decreased at endothelial cell-cell junctions (Figure 2D); and antibodies that recognized the cytoskeletal proteins vimentin (Figure 2E) and Twist (Figure 2F) were localized to the cytoplasm. The cells eventually reached confluence again for durations of 72 h and only VE-cadherin protein was expressed.

### Effect of rapamycin on the growth and migration of EA.hy926 cells

**Figure 3:**
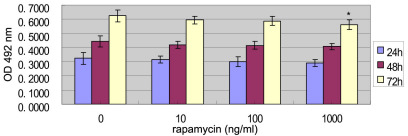
The effect of rapamycin on EA.hy926 cell growth. EA.hy926 cells were treated with 0 to 1,000 ng/ml rapamycin for 24, 48, or 72 h. Cell growth was determined by methyl thiazolyl tetrazolium assay (MTT). 0 ng/ml of rapamycin represents the control group. Bars represent standard deviation (SD; n=6 wells per measurement). Similar results were obtained in three independent experiments, and the data were shown as mean±SD. The asterisk indicates p<0.05, compared with the control group with independent samples *t*-test.

The growth of EA.hy926 cells was not affected by 10 ng/ml or 100 ng/ml rapamycin treatment for up to 72 h. In contrast, when the EA.hy926 cells were exposed to rapamycin for 72 h, the EA.hy926 cell growth was significantly inhibited by concentrations of 1000 ng/ml rapamycin (Figure 3).

**Figure 4:**
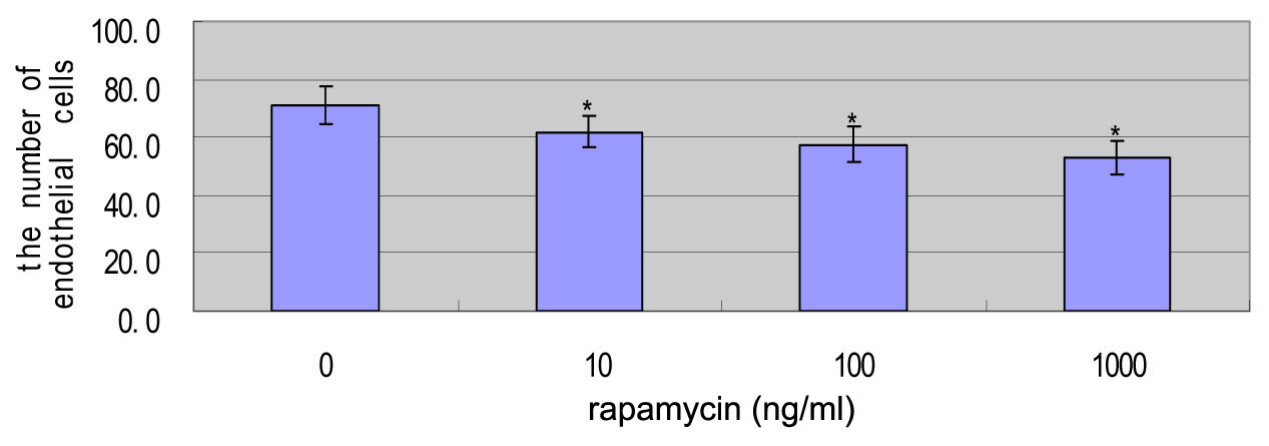
The effect of rapamycin on the migration of EA.hy926 cells. EA.hy926 cells were treated with 0 to 1,000 ng/ml rapamycin for 8 h. Cell migration ability was determined by the scratch wound assay method. 0 ng/ml of rapamycin represents the control group. Bars represent SD (n=6 photographs per measurement). Similar results were obtained in three independent experiments, and the data were shown as mean±SD. The asterisk indicates p<0.05, compared with the control group with independent samples *t*-test.

The effect of rapamycin on the migration of EA.hy926 cells was examined by using a scratch-wound assay. Rapamycin at concentrations of 10,100, and 1000 ng/ml inhibited the migration of EA.hy926 cells. There were statistically significant differences between the control group and the rapamycin groups (10, 100, 1000 ng/ml; Figure 4).

### Effect of rapamycin on gene expression cell marker proteins and Twist

**Figure 5:**
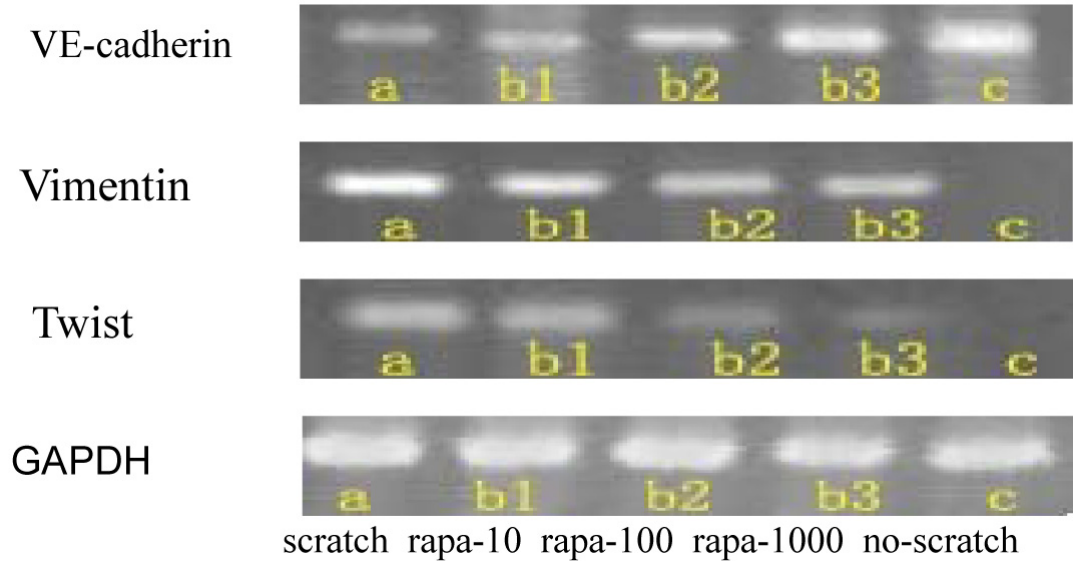
Reverse transcriptase–PCR gel electrophoresis images of vascular endothelial–cadherin/vimentin/Twist/glyceraldehyde 3-phosphate dehydrogenase mRNA expression in EA.hy926 cells. The effect of rapamycin on the gene expression of vascular endothelial (VE)-cadherin, vimentin, and Twist. (a, scratch group; b_1_, 10 ng/ml rapamycin group; b_2_, 100 ng/ml rapamycin group; b_3_, 1,000 ng/ml rapamycin group; c, no-scratch group).

**Figure 6:**
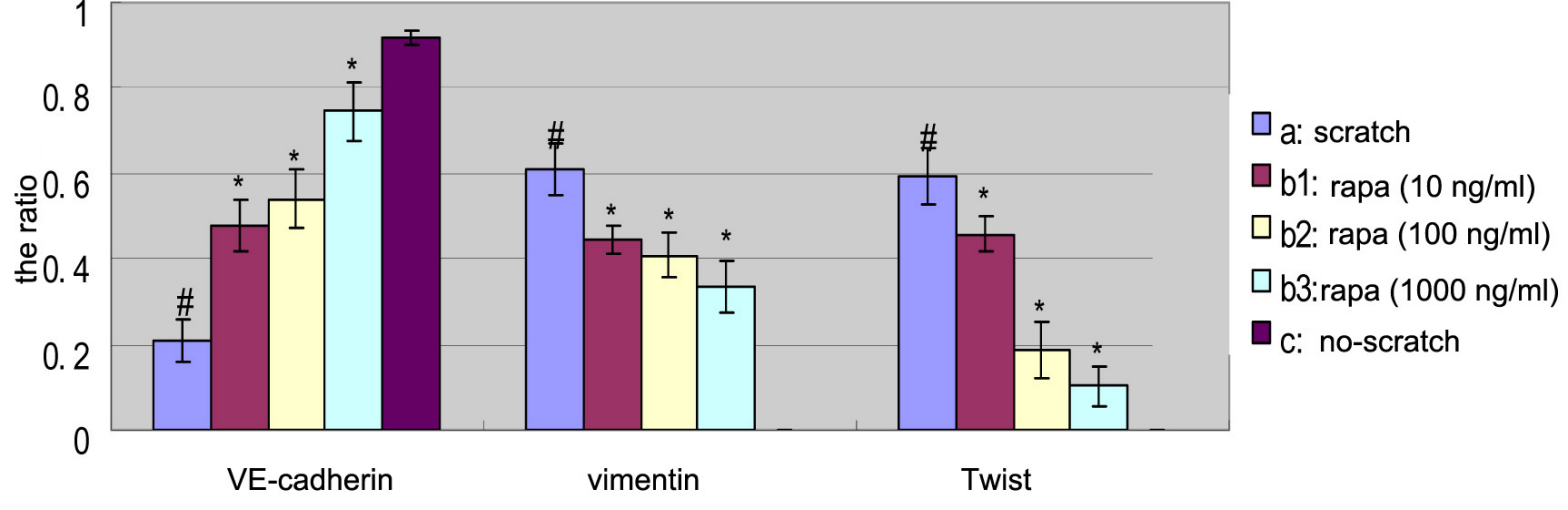
Relative expression ratio of the target gene versus the housekeeping gene (glyceraldehyde 3-phosphate dehydrogenase). The ratio for the vertical axis is relative expression ratio of the target gene versus the house-keeping gene (glyceraldehyde 3-phosphate dehydrogenase [*GAPDH*]). Three independent experiments were conducted. Bars represent standard deviation (SD), and the data were shown as mean±SD. The asterisk indicates p<0.05 in comparison between each rapamycin group and the scratch group with independent samples *t*-test. Pound sign (#) indicates p<0.05 in comparison between the scratch group and the no-scratch group with independent samples *t*-test. a, scratch group; b_1_, 10 ng/ml rapamycin group; b_2_, 100 ng/ml rapamycin group; b_3_, 1,000 ng/ml rapamycin group; c, no-scratch group.

After full cell confluency, there was only expression of VE-cadherin mRNA. After scratching, expression of VE-cadherin in the mRNA level downregulated, and EA.hy926 cells expressed Twist mRNA and vimentin mRNA, while 10, 100, and 1,000 ng/ml rapamycin inhibited the expression of Twist mRNA and vimentin mRNA. There were statistically significant differences between the no-scratch group and the scratch group and between the scratch group and the rapamycin groups (10, 100, and 1000 ng/ml) (Figure 5, Figure 6).

### Effect of rapamycin on matrix metalloproteinase-2 and matrix metalloproteinase-9 activity

**Figure 7:**
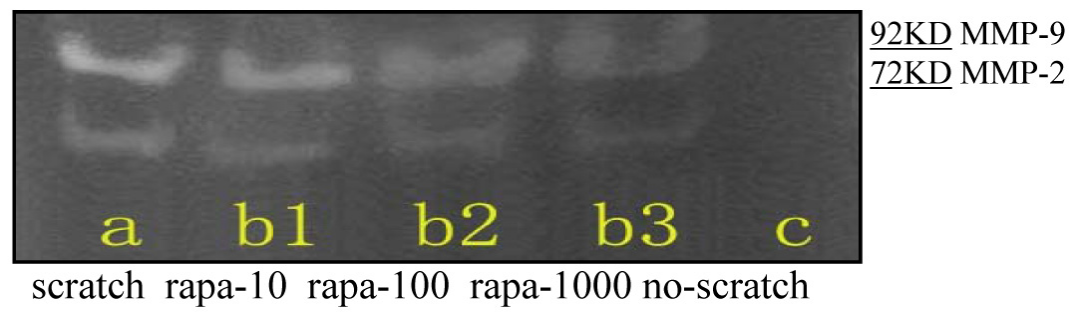
Gelatin zymography gel electrophoresis images of matrix metalloproteins 2 and 9 (a, scratch group; b_1_, 10 ng/ml rapamycin group; b_2_, 100 ng/ml rapamycin group; b_3_, 1,000 ng/ml rapamycin group; c, no-scratch group).

**Figure 8:**
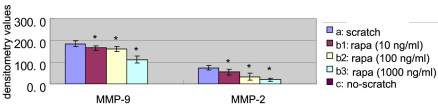
The result of densitometry values of MMP 2 and 9. The effect of rapamycin on the expression of matrix metalloprotein (MMP)-2 and −9 was determined by Gelatin zymography. Three independent experiments were conducted. Bars represent standard deviation (SD), and the data were shown as mean±SD. The asterisk (*) indicates p<0.05 in comparison between each rapamycin group and the scratch group with independent samples *t*-test. a, scratch group; b_1_, 10 ng/ml rapamycin group; b_2_, 100 ng/ml rapamycin group; b_3_, 1000 ng/ml rapamycin group; c, no-scratch group.

Results from the gelatin zymography assay showed that neither MMP-2 nor MMP-9 was detected in the culture supernatants of the no-scratch group (Figure 7), while there was expression of MMP-2 and MMP-9 protein in the scratch group and rapamycin groups. Both MMP-2 and MMP-9 were significantly reduced in EA.hy926 cells with a linear scratch wound with increasing concentration of rapamycin. There were statistically significant differences between the scratch group and the rapamycin groups (t=2.954, 5.640, 11.504, 3.056, 3.600, 9.349; p<0.05; Figure 7 and Figure 8).

## Discussion

Extracellular microenvironmental changes, such as hypoxia, result in increased expression of VEGF, which can activate endothelial cells. Activated endothelial cells can secrete protease (which dissolves vascular basement membranes) and extracellular matrix proteins, allowing capillary endothelial cells to migrate through their basement membrane into the extracellular matrix to form neovascular buds. This is the basic pathological process of angiogenesis. Rapamycin can effectively inhibit corneal neovascularization and tumor angiogenesis [4,7,19]. However, very little is known about rapamycin’s mechanism for inhibiting endothelial cell migration. This study aimed to fill this knowledge gap by observing rapamycin’s effects on EndoMT and the production of MMP-2 and MMP-9.

Numerous studies have shown that tumor cells originated in epithelial cells that transdifferentiated into mesenchymal cells, gained the ability to migrate, and promoted tumor metastasis [20]. When epithelial cells underwent mesenchymal transformation, their morphology changed from a cobblestone-like shape to a spindle-like shape [21,22]. This change in morphology was accompanied by decreased epithelial markers (E-cadherin) and increased Twist and mesenchymal markers (vimentin). Vimentin, a mesenchymal phenotype marker, was an intermediate filament cytoskeletal protein and was widely expressed in mesenchymal cells, which was very closely related to maintaining the fibroblasts’ shape and permitting cell migration. Vimentin was also expressed in HUVEC [23], which was more especially implicated in cellular motility and plasticity and was exclusively expressed in the migrating rat heart endothelial cell line [24].

The transcription factor Twist, a master regulator of embryonic morphogenesis, was recently identified as an important promoter of epithelial-to-mesenchymal transition (EMT) in breast cancers. In this process, Twist directly and indirectly caused the transcriptional repression of E-cadherin through the E-box elements on the E-cadherin promoter, thereby inducing vimentin expression [20]. Lopez et al. [25] has found that the tumor-induced upregulation of Twist repressed the activity of the human VE-cadherin promoter.

Endothelial cell migration plays an important role in angiogenesis [6]. Endothelial cells can change into mesenchymal cells during the embryonic development of the cardiovascular system [26,27]. In that process, endothelial cells express mesenchymal cell marker proteins and lead to a marked increase in endothelial migration. We found that VE-cadherin was mainly distributed at endothelial cell-cell junctions after cell confluency. However, VE-cadherin expression decreased significantly at endothelial cell-cell junctions and was mainly localized to the cytoplasm after receiving a scratch wound. At that point, vimentin and Twist were expressed in migrating endothelial cells. The cells eventually reached confluence again for durations of 72 h, exhibiting a cobblestone-like monolayer and expressing only the VE-cadherin protein. The results of this study demonstrated that endothelial cells can transform into mesenchymal cells, which can transition again into endothelial cells, and this process is reversible. We could conclude that Twist, which caused the transcriptional repression of VE-cadherin and induced the expression of vimentin, might also be an important factor in promoting EndoMT.

Rapamycin, isolated from a culture of *Streptomyces hygroscopicus*, is an immunosuppressant that specifically inhibits the mTOR in HUVEC [4]. MTOR is a serine/threonine protein kinase that plays a crucial role in cell growth, differentiation, proliferation, migration, and survival. Rapamycin can depress EMT induced by transforming growth factor-β, inhibit rabbit liver fibrosis, and reduce the kidney and peritoneal damage in the rat remnant kidney model by inhibiting the mTOR signaling pathway [28-30]. Rapamycin can inhibit VE-cadherin expression in endothelial cells by blocking mTOR [31]. This study found that the cell growth of EA.hy926 cells was significantly inhibited by 1000 ng/ml of rapamycin with 72 h exposure, but was not affected by short-term treatment up to 48 h or by concentrations of rapamycin lower than 1000 ng/ml. It was also observed that rapamycin at concentrations of 10, 100, and 1000 ng/ml for 8 h exposure successfully inhibited endothelial cell migration and vimentin and Twist expression. The results of this study demonstrate that rapamycin at concentrations of 10 and 100 ng/ml can effectively inhibit endothelial cell migration and suppress angiogenesis by depressing Twist expression, which could inhibit EndoMT without affecting cell growth.

Extracellular matrix degradation (ECM), which allows capillary endothelial cells to migrate through their basement membrane into the extravascular organization and provides the necessary space for new vessel formation, is an important process required during angiogenesis. MMPs are a class of endogenous proteolytic enzymes that are involved in ECM degradation. In particular, MMP-2 and MMP-9, which can degrade ECM and activate many growth factors, play an important role in tumor invasion and metastasis [32,33]. Rapamycin can suppress glioma invasion by blocking the production of MMP-2 and MMP-9 [13]. Endothelial cells produce MMPs at lower levels under normal circumstances, but endothelial cells activated by inflammation, trauma, and tumor growth can secrete a large number of MMPs that are activated quickly [34,35]. Some experimental studies have shown that normal corneal tissues produce MMPs at lower levels, which can be directly induced by some cytokines such as VEGF, fibroblast growth factor, and transforming growth factor and indirectly activated by active urokinase to promote angiogenesis in a disease state [4,36]. Kwon et al. found that rapamycin did not change MMP-9 expression in alkaline-burned corneal tissue [4], and this was possible because angiogenesis is a complex process regulated by multiple stimulatory and inhibitory factors in vivo. Some studies have found that HUVECs could produce MMP-2 and MMP-9 in an inactive form [37,38]. This experiment found that EA.hy926 cells secreted neither MMP-2 nor MMP-9 when they reached full confluence, but EA.hy926 cells activated by the scratch could produce MMP-2 and MMP-9 to increase cell migration. MMP-2 and MMP-9 were inhibited by 10, 100, and 1000 ng/ml doses of rapamycin to repress ECM degradation and cell migration. MMP-2 played an essential role in producing EMT [39]. Therefore, rapamycin might inhibit EndoMT by depressing MMP-2.

Our data indicate that Twist may also be an important factor in promoting EndoMT. The possible mechanism for the inhibition of angiogenesis by rapamycin is as follows: Rapamycin inhibited cell migration and extracellular matrix degradation by inhibiting EndoMT and endothelial cell secretion of MMP-2 and MMP-9. These are possible mechanisms for the inhibition of angiogenesis by Rapamycin.
